# Changes in duration of action of rocuronium following decrease in hepatic blood flow during pneumoperitoneum for laparoscopic gynaecological surgery

**DOI:** 10.1186/s12871-017-0335-1

**Published:** 2017-03-20

**Authors:** Yang Liu, Wen Cao, Yu Liu, Yun Wang, Ren Lang, Yun Yue, An-Shi Wu

**Affiliations:** 1 Department of Anesthesiology, Beijing Chaoyang Hospital, Capital Medical University, No. 8 Gongti South Road, Chaoyang District 100020 Beijing, China; 2 Department of Ultrasonography, Beijing Chaoyang Hospital, Capital Medical University, No. 8 Gongti South Road, Chaoyang District 100020 Beijing, China; 3 Department of Liver and Gallbladder, Beijing Chaoyang Hospital, Capital Medical University, No. 8 Gongti South Road, Chaoyang District 100020 Beijing, China

**Keywords:** Rocuronium, Hepatic blood flow, Pneumoperitoneum

## Abstract

**Background:**

A moderate insufflation pressure and deep neuromuscular blockade (NMB) have been recommended in laparoscopic surgery in consideration of the possible reduction in splanchnic perfusion due to the CO_2_-pneumoperitoneum. Since the liver is the major organ for rocuronium metabolism, the question of whether NMB of rocuronium would change with the variation of liver perfusion during pneumoperitoneum during laparoscopic surgery merits investigation.

**Methods:**

In this prospective study, a total of sixty female patients scheduled for either selective laparoscopic gynaecological surgery (group laparoscopy) or laparotomy for gynaecological surgery (group control) were analyzed. Rocuronium was administered with closed-loop feedback infusion system, which was also applied to monitor NMB complied with good clinical research practice (GCRP). The onset time, clinical duration, and recovery index were measured. Hepatic blood flow was assessed by laparoscopic intraoperative ultrasonography before insufflation/after entering the abdominal cavity (T1), 5 min after insufflation in the Trendelenburg position/5 min after skin incision (T2), 15 min after insufflation in the Trendelenburg position/15 min after skin incision (T3), 30 min after insufflation in the Trendelenburg position/30 min after skin incision (T4), and 5 min after deflation/before closing the abdomen (T5) in group laparoscopy/group control respectively. The relationship between the clinical duration of rocuronium and portal venous blood flow was analyzed using linear or quadratic regression.

**Result:**

The clinical duration and RI of rocuronium were both prolonged significantly in group laparoscopy (36.8 ± 8.3 min; 12.8 ± 5.5 min) compared to group control (29.0 ± 5.8 min; 9.8 ± 4.0 min) (*P* < 0.0001; *P* = 0.018). A significant decrease was found in portal venous blood flow during the entire pneumoperitoneum period in group laparoscopy compared with group control (*P* < 0.0001). There was a significant correlation between the clinical duration of rocuronium and portal venous blood flow (Y = 51.800-0.043X + (1.86E-005) *X* ^2^; r^2^ = 0.491; *P* < 0.0001).

**Conclusion:**

Rocuronium-induced NMB during laparoscopic gynaecological surgery might be prolonged due to the decrease in portal venous blood flow induced by CO_2_-pneumoperitoneum. Less rocuronium could be required to achieve a desirable NMB in laparoscopic gynaecological surgery.

**Trial registration:**

ChiCTR. Registry number: ChiCTR-OPN-15007524. Date of registration: December 4, 2015.

**Electronic supplementary material:**

The online version of this article (doi:10.1186/s12871-017-0335-1) contains supplementary material, which is available to authorized users.

## Background

Laparoscopic surgery has been widely used for the treatment of many gynaecological diseases on account of its minimally invasive technique [1]. To enlarge the intra-abdominal surgical space, the induction of pneumoperitoneum created by insufflation of carbon dioxide is necessary for laparoscopic procedures. However, several animal studies have demonstrated that high intra-abdominal pressure (IAP) induced by pneumoperitoneum may cause marked reduction in splanchnic and liver perfusion, and may even induce temporary impairment of liver function [2–5]. In humans, even brief periods of CO_2_ insufflation (45–60 min) have been shown to significantly reduce blood flow to organs within the peritoneal space [6]. Furthermore, patients undergoing laparoscopic surgery reacted with a transient elevation of liver enzymes, a reaction speculated to be attributable to impaired liver perfusion probably caused by IAP [7]. Thus, a moderate insufflation pressure (no more than 15 mmHg) has been recommended in laparoscopic gynaecological surgery [6, 8].

Another strategy to prevent events associated with high IAP is maintaining deep neuromuscular blockade (NMB) during the entire laparoscopic surgery. Rocuronium, an aminosteroid neuromuscular relaxant, is commonly used for anaesthesia in laparoscopic surgery. Deep NMB produced by rocuronium can improve surgical space conditions by increasing abdominal wall compliance during pneumoperitoneum and can accelerate bowel functional recovery postoperatively [9–11]. Since rocuronium is eliminated primarily through hepatic uptake and biliary excretion, the question of whether the duration of action of rocuronium is affected by decreased hepatic blood flow in response to CO_2_-pneumoperitoneum merits investigation. A previous study in cats reported that when hepatic inflow was considerably decreased via a portal vein-to-inferior vena cava shunt, the clinical duration of rocuronium increased almost threefold [12]. This result suggested that the pharmacodynamics of rocuronium should be correlated with hepatic perfusion.

The aim of the present study was to examine the duration of action of rocuronium during laparoscopic surgery and to further analyse the correlation between the rocuronium-induced NMB and decreased hepatic perfusion in response to pneumoperitoneum.

## Methods

This prospective, single center and nonrandomized clinical trial was approved by the Medical Ethics Committee of Chaoyang Hospital affiliated to the Capital Medical University (NO. 2015-KE-98) and registered in the Chinese Clinical Trial Registry (NO.ChiCTR-OPN-15007524) on 04 December, 2015. Prior written informed consent was obtained from all patients.

**Fig. 1:**
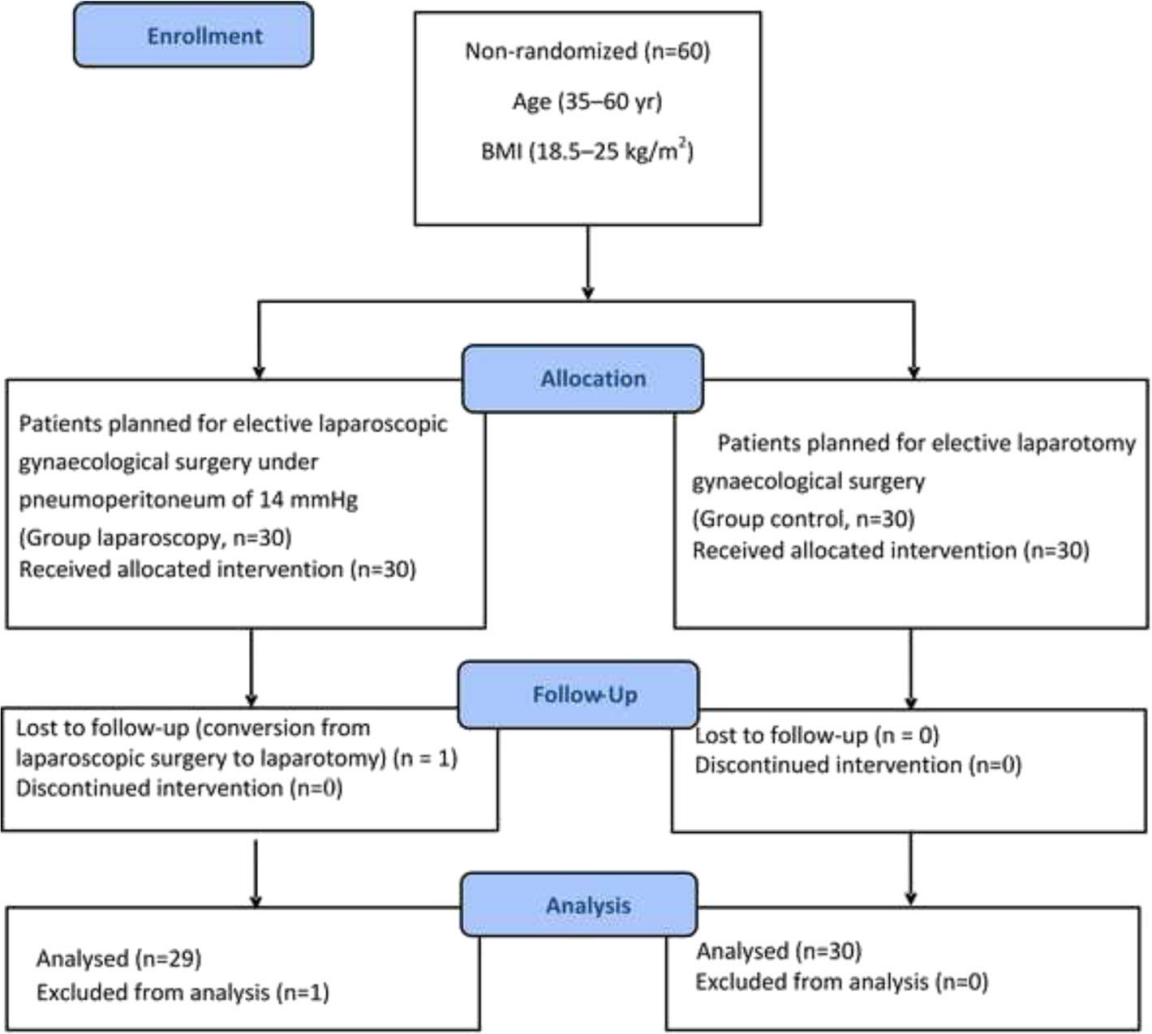
Flow-chart

A total of sixty female patients who were American Society of Anesthesiologists (ASA) physical statusI–II were enrolled in the study between December 2015 and May 2016. Among the 60 female patients, 30 underwent elective laparoscopic gynaecological surgery (group laparoscopy) and 30 underwent elective laparotomy gynaecological surgery (group control) concurrently. Considering the influence of age and body mass index (BMI) on the NMB of rocuronium [13, 14], subjects with age 35–60 years and with BMI 18.5 kg/m^2^ –25 kg/m^2^ were recruited. No patients were suffering from renal or hepatic disease, cardiovascular disease, diabetes, electrolyte disturbances, acid-base imbalances or neuromuscular disorders. Patients who were taking any medications known to interact with neuromuscular blocking agents (NMBAs), such as antibiotics, calcium-channel blockers, and anticonvulsants were also excluded [15]. Those who required supplemental muscle relaxants during laparoscopic surgery were excluded from the study. Durations of all of the gynaecological surgeries were limited to 2–3 h. The CONSORT Flow Diagram is reported in Fig. 1

### Anaesthesia techniques

Upon arrival in the operating room, an intravenous line containing Ringer’s lactate solution was inserted in the major left forearm of all the patients. Standard clinical monitoring included electrocardiograph, non-invasive arterial blood pressure, pulse oximetry and end-tidal carbon dioxide pressure (P_ET_CO_2_) and bispectral index (BIS, Aspect Medical Systems, Dublin, Ireland). All patients were administered total intravenous anaesthesia (TIVA) with propofol and remifentanil infusion at the plasma concentration of 2–4 μg/ml and 4–8 ng/ml respectively by target-controlled infusion (TCI) (Graseby 3500®, Diprifusor module, Watford, UK). The target plasma concentration of propofol was adjusted to maintain the BIS at about 40–60 until the end of surgery. All study subjects who received the adjunctive vasoactive drugs, such as esmolol, ephidrine which could influence the pharmacodynamics of rocoronium were excluded from the analysis.

### Neuromuscular block and monitoring by CLMRIS-I

The automatic infusion of rocuronium and the monitoring of NMB status were performed with the computerised Closed-Loop Muscle Relaxant Injection System (CLMRIS-I, Veryark, Nanning, Guangxi, China). The infusion rate of rocuronium was set on the basis of the anaesthesiologist’s clinical experience, and the monitoring of NMB was complied with good clinical research practice (GCRP) [16]. Two surface electrodes were placed over the ulnar nerve at the wrist and the acceleration transducers were attached to the thumb and index finger. The ulnar nerve was stimulated supramaximally with duration of 0.2 ms in a train-of-four (TOF) mode at a frequency of 2 Hz. The degree of NMB was defined as the ratio of the measurement of T1 in the TOF sequence to the corresponding control value, and was assessed every 20 s. The EMG responses (T1 and TOF ratio) were recorded and displayed on the monitoring screen of the CLMRIS-I. The temperature probe was placed over the skin of the adductor pollicis muscle, and skin temperature was maintained above 34 °C throughout the study period.

After the patients lost consciousness, the neuromuscular transmission monitoring of CLMRIS-I was initiated to calibrate and obtain a reference control value for all subsequent measurements. A bolus dose of 0.6 mg/kg rocuronium (Esmeron, Merck Frosst, Montreal, Canada) was then automatically infused, and tracheal intubation was performed when T1 of the TOF dropped to 0% of the control value. The patients were mechanically ventilated to maintain P_ET_CO_2_ 32–35 mmHg. To observe the clinical duration of rocuronium, the first feedback parameter of a single additional infusion was set at 25% recovery of T1. At this point, an additional bolus of rocuronium (30 μg · kg^−1^ · min^−1^) was injected, followed by a continuous infusion of 1.8 μg · kg^−1^ · min^−1^. Subsequently, the feedback threshold was changed to recovery of T1 to 15% for maintenance of a persistent and deep NMB. The infusion of rocuronium was stopped 30 min before closing the fascia and neostigmine, a muscle-relaxant antagonist, was permit to be administrated to the patients only after finishing the study of rocuroium. When TOF recovered to 90%, all patients underwent tracheal extubation. The anaesthetists were allowed to administer rocuronium manually or switch the closed-loop infusion to manual infusion of rocuronium during the operation, if needed, but any those subjects who had received these interventions were excluded from the analysis.

**Fig. 2:**
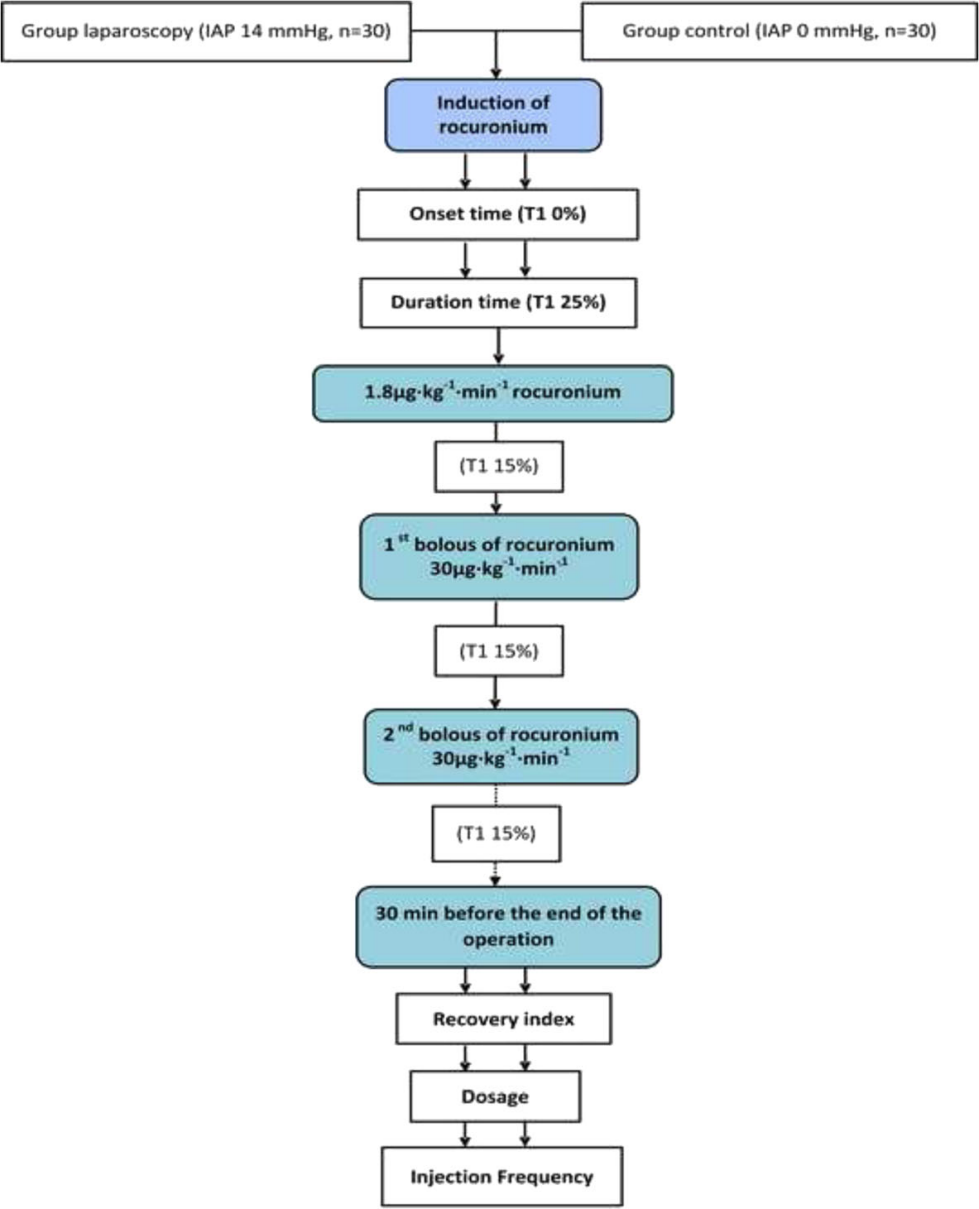
Trail profile

The following values for all patients were collected automatically by the CLMRIS-I [17], and the study flowchart is shown in Fig. 2:

1. ONSET TIME
2. CLINICAL DURATION
3. Infusion rate of rocuronium infused per unit time and weight (μg · kg^−1^ · h^−1^)
4. Bolus injection frequency of rocuronium during the whole operation per unit time
5. RECOVERY INDEX

### Laparoscopic procedures and ultrasonography

Following the induction of anaesthesia, CO_2_-pneumoperitoneum was established through one trocar at the umbilicus, and another two trocars were subsequently inserted. After a 7.5 MHz laparoscopic intraoperative ultrasonography (LIOU) probe (Aloka S-680, Aloka Medical Ltd, Tokyo, Japan) was introduced for measurement of hepatic blood flow, all CO_2_ introduced was allowed to escape. Colour flow mapping and pulse Doppler waveform of portal vein and hepatic artery trunk were performed using Aloka SSD-4000SV echograph to obtain the baseline value of hepatic flow before insufflation (T1). After this first measurement, pneumoperitoneum was reestablished at a pressure of 14 mmHg. Portal venous blood flow and Hepatic artery blood flow were achieved at 5 min after insufflation in the Trendelenburg position (T2), 15 min after insufflation in the Trendelenburg position (T3), 30 min after insufflation in the Trendelenburg position (T4), and 5 min after deflation (T5), respectively. In group control, the ultrasonic probe was inserted through the abdominal incision to measure portal venous blood flow and hepatic artery blood flow immediately after entering the abdominal cavity (T1), 5 min after skin incision (T2), 15 min after skin incision (T3), 30 min after skin incision (T4) and before closing the abdomen (T5) in open surgery. The following calculations were used to determine the haemodynamic parameters at each site:

(i) Vessel diameter (VD).
(ii) Cross-sectional area (CSA) = (VD/2)^2^ × π.
(iii) Mean flow velocity (MV).

Blood flow volume (BFV) = CSA × MV.

### Statistical analysis

Statistical analysis was performed using SPSS 19.0 (SPSS Inc., Chicago, IL, USA). A sample size calculation indicated that 25 patients in each group would be sufficient to find a statistically significant difference of 20% or more in the clinical duration between groups (0.05 two-sided significance level α = 0.05, 90% power β = 0.10). Categorical variables, expressed as numbers, were compared using the chi-squared test. Continuous variables, represented as means ± standard deviation (SD) were compared using the unpaired *t*-test or paired *t*-test of variance. The relationship between the clinical duration of rocuronium and the portal venous flow at differentiate IAP in group laparoscopy and group control at T4 was analyzed using linear or quadratic regression. A *P*-value < 0.05 was considered statistically significant.

## Results

From 2015-12-07 to 2016-5-13, we initially recruited 60 patients into the trial. Among the 60 included patients, one patient in group laparoscopy was excluded from the analysis due to conversion from laparoscopic surgery to laparotomy. Thus, 29 patients undergoing laparoscopic surgery and 30 patients undergoing open surgery were available for analysis. The laparoscopic and laparotomy gynaecologic surgeries were all performed successfully and no adjunctive vasoactive drugs were administered to the patients during the surgeries. Demographic and perioperative characteristics were similar between the two groups (Table 1).

**Table 1:** Characteristics of the patients

	Group control (*n* = 30)	Group laparoscopy (*n* = 29)
Age (yr)	46.2 ± 8.9	45.1 ± 7.3
Height (cm)	163.9 ± 2.1	162.8 ± 2.5
Body weight (kg)	62.6 ± 5.1	63.7 ± 7.6
BMI (kg/m^2^)	22.8 ± 1.7	23.2 ± 1.8
ASA classificationI/II(n)	19/11	16/13
Operation duration (min)	163.7 ± 16.4	157.6 ± 12.0
Infusion rate of propofol (μg · kg^−1^ · min^−1^)	88.6 ± 13.6	92.5 ± 16.1
Infusion rate of remifentanil (ng · kg^−1^ · min^−1^)	134.1 ± 47.5	128.9 ± 53.3

Data are presented as means ± standard deviation (SD) or number of patients

*BMI* body mass index, *ASA* American Society of Anesthesiologists

As demonstrated in Table 2, the onset time of rocuronium was similar between the two groups during the induction of anaethesia. Subsequently, however, the clinical duration of rocuronium and recovery index were prolonged significantly in group laparoscopy compared to group control (*P* = 0.018; *P* < 0.0001). Duing the entire operation, the supplement frequency and the infusion rate of rocuronium were lower in group laparoscopy than group control (*P* = 0.014; *P* = 0.046).

**Table 2:** Comparison of the effect of rocuronium in the two groups

	Group control (*n* = 30)	Group laparoscopy (*n* = 29)	*P*
Onset time (min)	95.5 ± 12.3	94.3 ± 12.7	0.708
Duration time (min)	29.0 ± 5.8	*36.8 ± 8.3* ^**^	<0.0001
Recovery index (min)	9.8 ± 4.0	*12.8 ± 5.5* ^*^	0.018
Bolus Frenquency	2.8 ± 0.7	*2.4 ± 0.6* ^*^	0.014
Infusion rate (μg · kg^−1^ · min^−1^)	6.3 ± 1.8	*5.4 ± 1.4* ^*^	0.046

Data are presented as means ± standard deviation (SD). ^*^ *P* < 0.05 compared with the control group. ^**^ *P* < 0.01 compared with control group

After calculations, the significant decrease in portal venous blood flow persisted over the entire study period compared with group control and the baseline value (*P* < 0.0001). Conversely, hepatic artery blood flow in group laparoscopy showed a statistically significant increase after the induction of pneumoperitoneum (*P* = 0.041). At 15 and 30 min after the initiation of Trendelenburg position (T3, T4), the blood flow volume (BFV) of hepatic artery gradually returned to close to the baseline value. At 5 min after deflation, the two groups did not differ in portal venous blood flow (*P* = 0.937) or hepatic artery blood flow (*P* = 0.902). Finally, the total hepatic blood flow during pneumoperitoneum at 14 mmHg was reduced to 58.27 ± 7.36%, 51.27 ± 6.26% and 50.94 ± 6.66% of the baseline value in the T2, T3 and T4 periods (not shown). In contrast, group control maintained a constant blood flow over the entire study period (Table 3).

**Table 3:** Parameters of hepatic blood flow in two groups assessed by laparoscopic intraoperative ultrasonography

			Group control (*n* = 30)	Group laparoscopy (*n* = 29)	*P*
Portal venous	T1	VD (cm)	0.99 ± 0.22	0.99 ± 0.21	0.971
		MV(cm/s)	24.02 ± 9.24	23.71 ± 7.25	0.888
		BFV (ml/min)	990.77 ± 133.65	1007.59 ± 145.36	0.645
	T2	VD (cm)	0.99 ± 0.23	*0.68 ± 0.18* ^** ##^	<0.0001
		MV (cm/s)	24.12 ± 9.82	21.81 ± 8.98	0.350
		BFV (ml/min)	991.50 ± 136.77	*432.90 ± 113.37* ^** ##^	<0.0001
	T3	VD (cm)	1.00 ± 0.24	*0.65 ± 0.18* ^**##^	<0.0001
		MV (cm/s)	23.91 ± 10.04	21.13 ± 6.55	0.214
		BFV (ml/min)	989.87 ± 135.42	*379.07 ± 101.42* ^** ##^	<0.0001
	T4	VD (cm)	0.99 ± 0.23	*0.62 ± 0.18* ^**##^	<0.0001
		MV (cm/s)	23.79 ± 9.39	23. 08 ± 6.31	0.733
		BFV (ml/min)	987.03 ± 107.15	*389.38 ± 106.68* ^** ##^	0.001
	T5	VD (cm)	1.00 ± 0.23	0.98 ± 0.22	0.806
		MV (cm/s)	23.63 ± 9.61	23.81 ± 7.59	0.937
		BFV (ml/min)	995.13 ± 128.33	997.97 ± 144.60	0.937
Hepatic artery	T1	VD (cm)	0.42 ± 0.12	0.42 ± 0.13	0.945
		MV (cm/s)	37.79 ± 12.93	37.18 ± 13.07	0.856
		BFV (ml/min)	275.43 ± 77.73	273.62 ± 79.32	0.930
	T2	VD (cm)	0.42 ± 0.12	*0.35 ± 0.10* ^* ##^	0.029
		MV (cm/s)	37.69 ± 13.55	*60.88 ± 18.11* ^* *##^	<0.0001
		BFV (ml/min)	273.80 ± 76.33	*320.31 ± 93.93* ^* ##^	0.041
	T3	VD (cm)	0.42 ± 0.12	0.38 ± 0.12	0.238
		MV (cm/s)	37.43 ± 13.30	*46.89 ± 15.72* ^*^	0.015
		BFV (ml/min)	273.33 ± 77.88	287.83 ± 87.37	0.504
	T4	VD (cm)	0.41 ± 0.12	0.41 ± 0.13	0.919
		MV (cm/s)	37.39 ± 12.05	38.05 ± 12.56	0.837
		BFV (ml/min)	274.50 ± 81.28	274.72 ± 83.69	0.992
	T5	VD (cm)	0.42 ± 0.12	0.42 ± 0.13	0.952
		MV (cm/s)	37.14 ± 12.01	36.77 ± 12.93	0.909
		BFV (ml/min)	276.23 ± 80.66	273.62 ± 81.20	0.902
Total Hepatic blood flow	T1	BFV (ml/min)	1266.20 ± 207.16	1281.21 ± 218.34	0.787
	T2	BFV (ml/min)	1265.30 ± 207.88	*753.21 ± 194.84* ^** ##^	<0.0001
	T3	BFV (ml/min)	1263.20 ± 208.19	*666.90 ± 179.72* ^**##^	<0.0001
	T4	BFV (ml/min)	1261.53 ± 184.02	*664.10 ± 185.95* ^**##^	<0.0001
	T5	BFV (ml/min)	1271.37 ± 203.12	1271.59 ± 219.67	0.997

Data are presented as means ± standard deviation (SD). ^*^ *P* < 0.05 compared with the control group. ^**^ *P* < 0.01 compared with the control group. ^#^ *P* < 0.05 compared with before pneumoperitoneum period. ^##^ *P* < 0.01 compared with before pneumoperitoneum period

*VD* vessel diameter, *MV* mean flow velocity, *BFV* blood flow volume

**Fig. 3:**
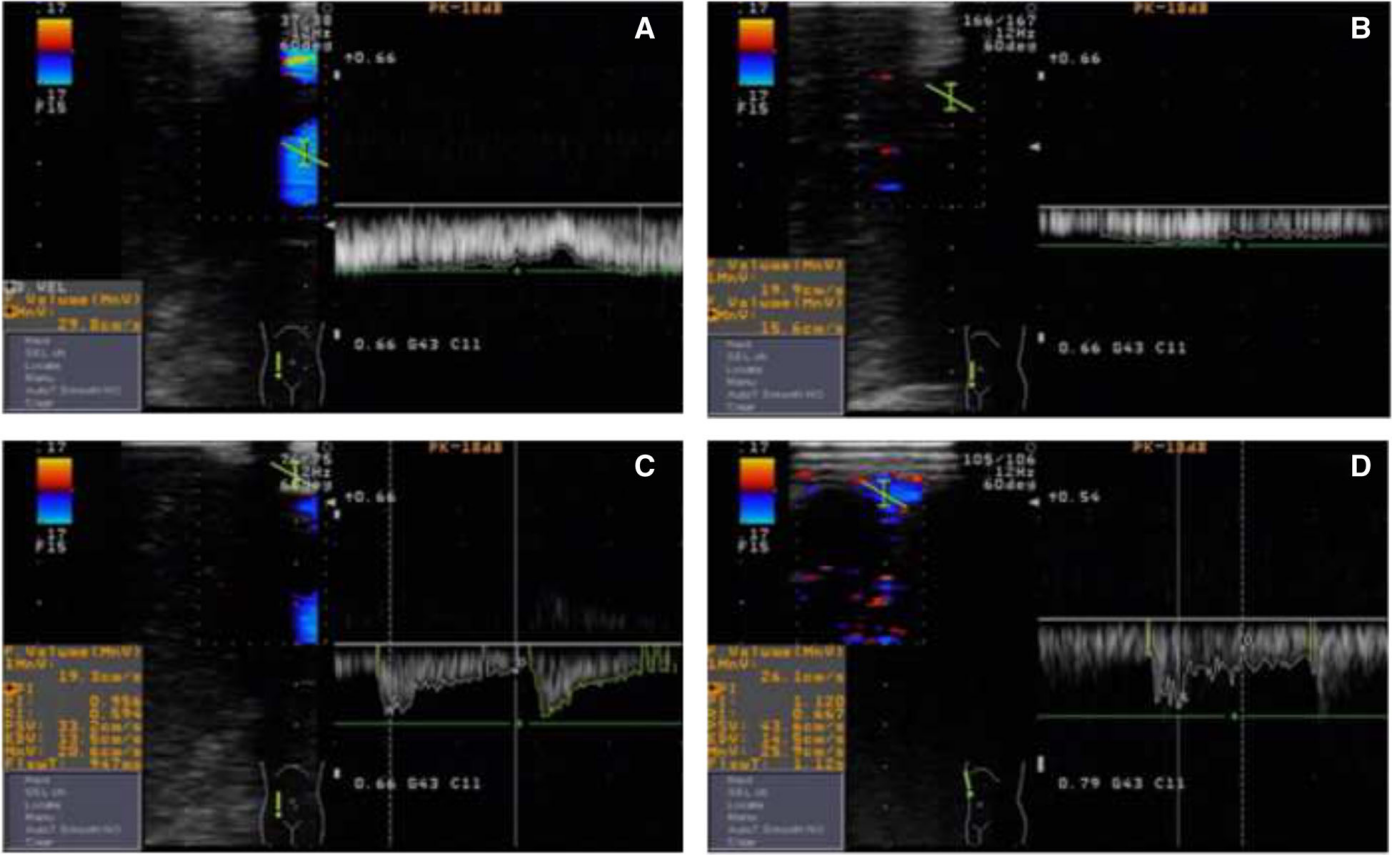
Morphologic changes in the hepatic blood flow induced by CO_2_-pneumoperitoneum on laparoscopic intraoperative ultrasonography. **a** Two-dimensional image of portal vein before insufflation of CO_2_. **b** Thirty min after insufflation of CO_2_ in the Trendelenburg position, the portal vein showed a marked decrease in the VD from 0.90 cm to 0.64 cm, as well as a marked decrease in MV from 29.50 cm/s to 14.60 cm/s. The calculated BFV decreased from 1126 ml/min to 281 ml/min. **c** Two-dimensional image of hepatic artery before insufflation of CO_2_. **d** Thirty min after insufflation of CO_2_ in the Trendelenburg position, the MV of hepatic artery was significantly increased from 19.2 cm/s to 26.1 cm/s with the VD less affected. The calculated BFV was similar (93 ml/min vs 96 ml/min) to that before insufflation of CO_2_. *VD* vessel diameter, *MV* mean flow velocity, *BFV* blood flow volume

An example of the pulsed wave Doppler ultrasonograph is shown in Fig. 3 as a representative graph of the changes in the vessel diameter (VD) and the mean flow velocity (MV) of hepatic vessels assessed by the LIOU before pneumoperitoneum and 30 min after insufflation of CO_2_ in the Trendelenburg position.

**Fig. 4:**
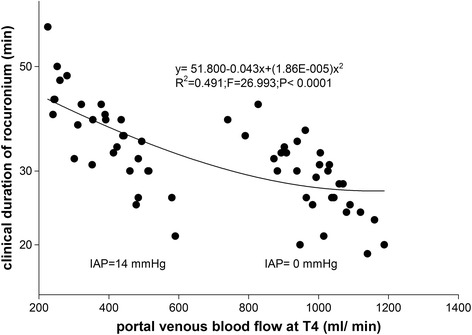
Quadratic correlation between portal venous blood flow at T4 and clinical duration of rocuronium

In quadratic regression models, total 59 subjects in two groups exhibited a significant correlation between the clinical duration and portal venous blood flow in the quadratic regression analysis (Y = 51.800-0.043X + (1.86E-005) *X* ^2^; Y: Duration; X: Portal venous blood flow; r^2^ = 0.491; F = 26.993; *P* < 0.0001). As shown in Fig. 4, the duration of action of rocuronium was prolonged with the decrease in the portal venous blood flow. According to the quadratic formula, the clinical duration of rocuronium would reach its minimum as portal venous blood flow increased to its upper limit. The longest duration of action of rocuronium was no more than 51.8 min, when portal venous blood flow decreased to almost zero.

## Discussion

There are three major findings in the present study. First, CO_2_-pneumoperitoneum at 14 mmHg in the Trendelenburg position induced a significantly long clinical duration of rocuronium. Second, hepatic portal venous blood flow decreased significantly in patients undergoing laparoscopic surgery compared to those undergoing conventional open surgery. Third, the prolongation of the clinical duration of rocuronium correlated with the decrease in hepatic portal venous blood flow in response to CO_2_-pneumoperitoneum.

Several reports have suggested that deep NMB was essential for improving surgical conditions without decreasing perfusion of the internal organs during pneumoperitoneum [9, 10]. Conversely, Paek CM et al. demonstrated that a single intubating dose of rocuronium could satisfy the requirements of laparoscopic pelvic surgery [18]. To the authors’ knowledge, the present study is the first to investigate the pharmacodynamics of rocuronium throughout laparoscopic gynaecological surgery. During the entire surgery, rocuronium was administered with CLMRIS-I, a closed-loop infusion system. It had the dual functions of both monitoring NMB as well as delivering automatic feedback infusion of rocuronium. TOF ratio could be used to help regulating the infusion of rocuronium automatically, which could provide stable and uniform muscle relaxation. The findings of pharmacodynamics of muscle relaxants have shown that the duration of action of rocuronium was prolonged significantly during the laparoscopic surgery compared to laparotomy surgery. In addition, a slower recovery of neuromuscular function was observed in laparoscopic surgery than in laparotomy surgery. These variabilities in NMB may be due to the decrease in hepatic perfusion in response to pneumoperitoneum. Portal venous blood flow accounts for about two-thirds of the total blood flow to the liver, and the clear correlation between the duration of action of rocuronium and portal venous blood flow was found in the present study, which confirmed our previous hypothesis that the prolongation of NMB of rocuronium might be attributable to the depression of hepatic perfusion during the lapaoscopic surgery and portal venous blood flow might have a predominant influence on the time course of action of rocuronium.

There has been controversy regarding the effect of CO_2_-pneumoperitoneum on decrease in hepatic perfusion [19, 20], which may be explained as follows: First, different extents of IAP by pneumoperitoneum and operative positions of the patients should be considered. In the previous studies, the opinions that the changes in hepatic blood flow were IAP-related have been remained consistent, while the conclusions regarding the thresholds at which IAP reduced the hepatic blood flow were not in agreement. The operative position, especially Trendelenburg in contrast to the supine and supine-lithotomy, might be associated with the decreases in hepatic perfusion resulting from the physiological response to increased IAP [21, 22]. Second, TEE has limitations for the assessment of hepatic venous blood flow as the angle of insonation is more than 60°. By contrast, LIOU-based measurements of the portal venous and hepatic artery blood flow are reliable and accurate owing to direct placement of probes closer to the target vessels. Third, blood flows in the hepatic artery and portal vein have different responses to elevated IAP caused by CO_2_-pneumoperitoneum. The portal venous blood flow was decreased significantly with the decrease of venous diameter at 14 mmHg of abdominal pressure. However, hepatic artery blood flow was selectively preserved to maintain the liver blood supply during laparoscopy-associated portal venous blood flow reduction, which might provide protection for liver function during CO_2_-pneumoperitoneum [22, 23]. However, this increase in hepatic artery blood flow in virtue of the increased flow rate has not remained long in the present study during sustained elevations in IAP.

We acknowledge the limitations of this study. First, the study was not randomised or blinded. The close matching of the two groups could reduce potential bias in the analysis of the primary outcome. Second, we did not directly measure the plasma concentrations of rocuronium to clarify the elimination of rocuronium. CLMRIS-I used in our study was an automated delivery system based on pharmacodynamic, but not pharmacokinetic parameters of NMBAs.

## Conclusions

In conclusion, this study indicated that the duration of action of rocuronium might be prolonged due to the decrease in portal venous blood flow induced by CO_2_-pneumoperitoneum. Therefore, less rocuronium could meet the requirement of achieving a desirable NMB in laparoscopic gynaecological surgery. Meanwhile, neuromuscular monitoring is worth considering so that residual action of rocuronium could be avoided in laparoscopic gynaecological surgery.

## Additional files

Additional file 1: Pharmacodynamics of rocuronium in two groups. Onset time, duration time, recovery index, bolous frequency and indusion rate of rocuronium. (XLSX 13 kb)
Additional file 2: Parameters of hepatic blood flow in two groups. The vessel diameter, mean flow velocity and blood flow volume of hepatic blood flow. (XLSX 27 kb)

## Abbreviations

ASA: American Society of Anesthesiologists
BFV: Blood flow volume
BMI: Body mass index
CLMRIS-I: Closed-loop muscle relaxant injection system
GCRP: Good clinical research practice
IAP: Intra-abdominal pressure
LIOU: Laparoscopic intraoperative ultrasonography
MV: Mean flow velocity
NMB: Neuromuscular blockade
NMBAs: Neuromuscular blocking agents
P_ET_CO_2_: End- tidal carbon dioxide pressure
RI: Recovery index
TCI: Target-controlled infusion
TIVA: Total intravenous anaesthesia
TOF: Train- of-four
VD: Vessel diameter
